# Snapshots of a Non-Canonical RdRP in Action

**DOI:** 10.3390/v13071260

**Published:** 2021-06-28

**Authors:** Diego S. Ferrero, Michela Falqui, Nuria Verdaguer

**Affiliations:** Institut de Biología Molecular de Barcelona, CSIC, Parc Científic de Barcelona, Baldiri Reixac 15, 08028 Barcelona, Spain; michelafalqui@gmail.com

**Keywords:** permuted RNA-dependent RNA polymerase, permutatetravirus, replication-elongation complexes, RNA virus replication, structural studies

## Abstract

RNA viruses typically encode their own RNA-dependent RNA polymerase (RdRP) to ensure genome replication and transcription. The closed “right hand” architecture of RdRPs encircles seven conserved structural motifs (A to G) that regulate the polymerization activity. The four palm motifs, arranged in the sequential order A to D, are common to all known template dependent polynucleotide polymerases, with motifs A and C containing the catalytic aspartic acid residues. Exceptions to this design have been reported in members of the Permutotetraviridae and Birnaviridae families of positive single stranded (+ss) and double-stranded (ds) RNA viruses, respectively. In these enzymes, motif C is located upstream of motif A, displaying a permuted C–A–B–D connectivity. Here we study the details of the replication elongation process in the non-canonical RdRP of the *Thosea asigna* virus (TaV), an insect virus from the Permutatetraviridae family. We report the X-ray structures of three replicative complexes of the TaV polymerase obtained with an RNA template-primer in the absence and in the presence of incoming rNTPs. The structures captured different replication events and allowed to define the critical interactions involved in: (i) the positioning of the acceptor base of the template strand, (ii) the positioning of the 3’-OH group of the primer nucleotide during RNA replication and (iii) the recognition and positioning of the incoming nucleotide. Structural comparisons unveiled a closure of the active site on the RNA template-primer binding, before rNTP entry. This conformational rearrangement that also includes the repositioning of the motif A aspartate for the catalytic reaction to take place is maintained on rNTP and metal ion binding and after nucleotide incorporation, before translocation.

## 1. Introduction

RNA viruses include the most abundant group of human, animal and insect pathogens. These viruses encode a specialized enzyme, the RNA-dependent RNA polymerase (RdRP), responsible for RNA genome replication and transcription. All known RdRPs share a similar closed right-hand architecture, with the fingers, palm and thumb subdomains and use the same mechanism of catalysis [1]. This has led to the hypothesis that all these enzymes share a common origin [2].

RdRPs function either as single polypeptides or in a complex with other viral or host components to transcribe and replicate the viral RNA genome. They use a variety of strategies to recognize substrates and coordinate the different steps of RNA synthesis [3].

The RdRP closed hand conformation encircles seven conserved structural and functional motifs (A to G) that contribute to different steps of the RdRP activity. Four of them (A to D) are common to all known template-dependent polynucleotide polymerases and are located in the palm subdomain. The palm consists of four antiparallel β-strands, flanked by three α-helices. Motif A is located at the end of a β-strand in the central β-core and motif C is at the top of the contiguous β-hairpin. Both motifs A and C contain the strictly conserved aspartates that coordinate the catalytic Mg^2+^ ions that are crucial for catalysis and pyrophosphate release [1,3]. Moreover, a basic residue of motif D would play the role of the proton donor to the leaving pyrophosphate determining the efficiency and fidelity of nucleotide addition [4].

Exceptions to the canonical A–B–C–D organization have been reported for RdRPs in members of the Permutotetraviridae and Birnaviridae families of positive single stranded (+ss) and double stranded (ds) RNA viruses, respectively. In these enzymes, motif C is located upstream of motif A forming a noncanonical C–A–B–D arrangement with a unique connectivity of the major structural elements of the active site [5,6]. However, the crystal structures of permuted RdRPs from representatives of the Permutotetraviridae and Birnaviridae families revealed that in spite of their permuted connectivity, the overall architecture of their catalytic sites is identical to those of canonical RdRPs [7,8,9,10].

*Thosea asigna* virus (TaV) is a (+) ssRNA virus that infects the larvae of a defoliating moth (*Setothosea asigna*) and the prototypic member of the Permutotetraviridae family [11]. In a previous work we have structurally and functionally characterized the non-canonical RdRP domain of TaV (TaV_pol_: residues 1-674 of the ORF1 product), showing close resemblances with flaviviral RdRPs. In particular, the presence of a long priming loop, protruding into the active cavity that is known to serve as a support platform for the de novo initiation of RNA synthesis. In addition, in vitro polymerization assays showed that the TaVpol was active in a primer-independent reaction and exhibited a kinetics profile very similar to those observed for flaviviral RdRPs. Altogether these results indicated the existence of an unexpected evolutionary link between permutatetravirus and flavivirus polymerases [7]. The crystal structure of TaV_pol_ was solved in its apo- form and bound to different rNTP substrates. In all crystal forms analyzed the protein was arranged in dimers, stabilized by mutual interactions between the flexible N-terminal arm of one molecule and the active site cleft of its interacting neighbor. A putative model of the TaV replication-initiation, generated by the structural superimposition of the ϕ6 polymerase replication-initiation complex [12] onto the TaV_pol_ active site, positioned the basic residue R12 in close contact with the phosphate moiety of the templating (t+1) nucleotide. This model prompted one to hypothesize that the N-terminal arm in the first steps of the de novo replication process has a regulatory role [7].

Here we report the X-ray structures of TaV_pol_ captured in three replication-elongation states, revealing high resolution mechanistic details of different events in the nucleotide addition cycle for a non-canonical RdRP. In addition, the structural characterization of a new crystal form of TaVpol in its unbound-state showed a large reorganization of the polymerase motif F that is moved about 20 Å from its expected position, forming the ceiling of the NTP tunnel, towards the catalytic cavity. Similar conformational rearrangements of motif F were observed in the RdRPs of the flaviviruses JEV [13] and ZIKV [14].

## 2. Materials and Methods

### 2.1. Chemical Synthesis

The RNA oligonucleotide used for the crystallization studies, 5′-CAAAAUUU-3′ (purified by PAGE and dry), was purchased from Sigma. Natural rNTPs and non-hydrolysable UTP (uridine-5’-[(α,β)-imido]triphosphate) were obtained from Jena bioscience.

### 2.2. DNA Constructs

cDNA from TaV ORF1 was obtained from Genescript as previously described [7]. The DNA sequence codifying for the amino-terminal RdRP domain of TaV (residues 10-673) was amplified by PCR using Phusion (ThermoFisher Scientific) and Fw and Rv primers flanked by BamHI (5′ CCCGGATCCACCCGTCTCTCCTTGGAAGC 3′) and HindIII (5′ CCCAAGCTTGATGACCACGCGAGTGTCCATG 3′) restriction sites, respectively, and a CCC 5′ tail. The obtained fragment was purified, digested and cloned into a modified version of the pACYCDuet vector (Millipore) previously cleaved with identical enzymes (pACYCDuet-TaV_pol_-Δ10). Vector modification consisted of the addition of the coding sequence for the Tobacco etch virus (TEV) protease site downstream of the sequence coding for the His tag, in a BamHI site. The resulting recombinant protein contained a removable hexa-histidine tag at the protein N-terminus.

Deletion in the priming loop (amino acids 607-624) was performed by PCR in a 50 µL reaction, using a site-directed mutagenesis protocol previously described [15], in which forward and reverse primers (5′TCAGACGGCTACCCTTCCTACGACTGGG3′ and 5′ACCTCTGCCACCTCCCTCCTGGAC3′, respectively) were used to amplify the plasmid containing the sequence of interest. The blunt-ended PCR-generated DNA was phosphorylated and ligated by T4 polynucleotide kinase (T4 PNK) and T4 DNA ligase treatments, respectively, following the manufacturer’s instructions (New England BioLabs). Finally, 1 µL of DpnI (Thermo Fisher) was added and incubated during 1 h at 37 °C to remove the template DNA and used to transform *E. coli* (DE3). The presence of the designed deletion in the final plasmid (pACYCDuet-Δ10-TaV_pol_-Δ607-624) was confirmed by DNA sequencing.

### 2.3. Protein Expression and Purification

The recombinant plasmids to produce the TaV_pol_ versions, Δ10-TaV_pol_ and Δ10-TaV_pol_-Δ607-624, were transformed into the *E. coli* BL21 (DE3) strain and cultured at 37 °C in LB media containing 35 µg/mL of chloramphenicol until OD600 = 0.7, induced with 0.1 mM isopropyl-ß-D-1-thiogalactopyranoside (IPTG) and incubated ON at 22 °C. Cells were harvested by centrifugation (JLA-9.1000 rotor, 3500 rpm, 30 min) and the bacterial pellet was resuspended in lysis buffer or buffer A (50 mM Tris pH 7.5, 500 mM NaCl, 5% glycerol and 2 mM DTT) supplemented with a protease inhibitor cocktail (Complete EDTA-Free; Roche), 10 µg/mL of DNAse (Bio Basic, Canada Inc., Markham, ON, Canda), 10 µg/mL of RNAse (Neo-Biotech), 0.1 mg/mL of lysozyme and 5 mM MgCl2. Cells were homogenized at 4 °C on a cell disrupter at 1.4 MPa (Constant Cell Disruption Systems, UK) and the extract clarified by centrifugation at 48,000*g* for 30 min in a JA25.50 rotor (Beckman Coulter) at 4 °C. The supernatant was filtered (0.22 µm, Millipore) and loaded onto a buffer A pre-equilibrated 5 mL His-trap HP column (GE Healthcare) with 0.5 mL/min flux. The column was washed with 10 column volumes (CV) of lysis buffer without additives, mixed with 20% of buffer B (50 mM Tris pH 7.5, 500 mM NaCl, 5% *v*/*v* glycerol, 2 mM DTT and 500 mM imidazole) and eluted with a linear gradient of 30 CV until 100% B.

Fractions containing the protein of interest were pooled and dialyzed ON at 4 °C against buffer 50 mM Tris pH 7.5, 300 mM NaCl, 5% *v*/*v* glycerol and 2 mM DTT. The dialyzed protein was subsequently treated with TEV protease (0.02 mg/mL) ON at 4 °C, then incubated with TALON resin (Takara) for 1 h at 4 °C and centrifuged to eliminate TEV. The supernatant was further purified by size-exclusion chromatography on a Superdex 200 Increase 10/300 column (GE Healthcare), exchanging the buffer to 50 mM MES pH6, 250 mM NaCl, 5 mM DTT and 5% *v*/*v* glycerol. Finally, fractions containing the purified protein were pooled and concentrated using Amicon Ultra 10K filters (Millipore) to get 40 mg/mL.

### 2.4. Crystallization

The initial crystallization trials of Δ10-TaV_pol_ and Δ10-TaV_pol_-Δ607-624 were performed by the sitting-drop vapor diffusion method at 293 K with a 200 nL drop (1:1 ratio) in 96-well Greiner plates, using a nanoliter-drop dispenser robot (Phoenix, ARI) and several screens (Crystal Screen 1, Crystal Screen 2, Wizard I, Wizard II, SaltRX, JBScreen Basic, JBS Classic HTS, Natrix, Protein-nucleic acid complex crystal screen, TOP96) available at the automated crystallography platform (PAC) (IBMB-CSIC, Barcelona). Crystals appeared after 1 day, in different conditions. Several rounds of optimization conditions (protein and precipitant concentrations and drop size) were performed to obtain suitable crystals for X-ray studies. The best Δ10-TaV_pol_ crystals were obtained in 19% PEG 3350, 0.18 M MgSO_4_. The Δ10-TaV_pol_-Δ607-624 construct (unbound-form) was crystallized in 0.1 M sodium acetate pH 4.6, 0.25 M (NH_4_)SO_4_ and 26% PEG4000. The Δ10-TaV_pol_-Δ607-624-RNA complex was obtained and crystallized as follows: The partially autocomplementary RNA oligonucleotide 5′CAAAAUUU3′ was resuspended in water (previously treated with 0.1% *v*/*v* DEPC) at a 4 mM concentration and annealed by heating at 90 °C during 1 min and slowly cooling to 10 °C at a rate of 1 °C/min on a PCR machine. The dsRNA molecule was then added to the Δ10-TaVpol-Δ607-624 protein solution in an equimolar proportion. The nucleotides UTP or a mix of UTP and GTP were also added at 2 mM concentration and the solution was incubated, for 2 h at 4 °C before starting the crystallization trials. Well diffracting crystals of the TaV_pol_-RNA complex were obtained in two days from a solution, containing 0.1 M sodium acetate pH 4.6, 0.22 M (NH_4_)SO_4_ and 28% PEG2000MME. These crystals were soaked with 2 mM MgCl_2_ for a few minutes in the cryobuffer solution (crystallization buffer in a 20% (*v*/*v*) glycerol) to obtain the ternary Δ10-TaV_pol_-Δ607-624-RNA-UTP and Δ10-TaV_pol_-Δ607-624-RNA–uridine-5’-[(α,β)-imido]triphosphate complexes.

All crystal optimization experiments were performed at 293 K, by sitting drop vapor diffusion, in an MRC 48 well plate using a 200 µL reservoir of the crystallization buffer and a 1 µL drop (1:1 ratio).

### 2.5. X-Ray Data Collection, Processing, Structure Solution and Refinement

Crystals were harvested in cryo-loops (Molecular Dimensions), soaked for 1 min in a reservoir solution and 20% (*v*/*v*) glycerol and flash-frozen in liquid nitrogen. Diffraction data were collected at 100 K on the BL13-XALOC beamline at the ALBA Synchrotron (Cerdanyola del Vallès, Spain) and the P13 and P14 beamlines, operated by EMBL at the PETRA III storage ring (DESY, Hamburg, Germany).

Diffraction data were processed using XDS [16]. Space group determination and data reduction were performed with POINTLESS/AIMLESS [17]. Data collection statistics are shown in Table 1. Structures were solved by molecular replacement with program MOLREP [18], using the structure of the TaV_pol_ apo-form (PDB: 4XHI) as a search model. Refinement was done alternating cycles of manual rebuilding using Coot [19] and automatic refinement using Phenix [20]. Data refinement statistics are listed in Table 1. Model geometry was validated using Molprobity [21]. Illustrations were prepared with Chimera [22]. A video of morphing conformations of TaV_pol_ was performed with Chimera using the linear interpolation method (30 interpolation steps) between coordinates of Δ10-TaV_pol_ Mg^2+^ (PDB: 7OM2), Δ10-TaV_pol_ Δ607-624-RNA (PBB: 7OM6) and Δ10-TaV_pol_ Δ607-624-RNA-UTP (PDB:7OM9). Catalytic and non-catalytic Mg^2+^ ions and PPi were shown.

## 3. Results

### 3.1. Design of the Crystallizable Catalytic Complexes of TaV_pol_

Initial approaches to obtain the crystallizable replication elongation complexes of TaV_pol_ failed, despite using template-primer RNAs of different lengths and sequences. The structures of TaV_pol_ previously characterized [7] indicated that the priming loop λ6 should be reorganized to facilitate the opening of the central cavity to accommodate the growing dsRNA product. A number of crystallizable binary (RdRP-RNA) and ternary (RdRP-RNA/rNTP) complexes of the HCV RdRP NS5B have been previously obtained after modifying an equivalent priming element, the β-hairpin, protruding from the NS5B thumb subdomain [23,24]. Following a similar strategy, we modified the TaV_pol_ λ6 loop, eliminating residues from A607-I624 and adding a G–G dipeptide as a linker between G606 and S625 to generate the TaV_pol_Δ607-624 construct. In addition, the proximity of the N-terminal arm of the neighboring polymerase to λ6, and to the polymerase template channel suggested a key switching role of the N-terminus in the initiation-to-elongation transition. The first ten residues, not visible in the electron density maps of the previously determined structures [7], appeared to disturb important protein–RNA interactions and was eliminated to increase the stability of RNA binding.

The final designed construct, Δ10-TaV_pol_Δ607-624, was produced, purified and crystallized under three different conditions (see the Materials and Methods section): (i) bound to a template/primer RNA, (ii) bound to a template/primer RNA in the presence of a non-hydrolysable UTP (uridine-5’-[(α,β)-imido]triphosphate) and (iii) bound to a template/primer RNA, in the presence of a UTP/GTP mixture (Table 1).

### 3.2. The Structure of Δ10-TaV_pol_Δ607-624 Bound to a Template-Primer RNA

**Figure 1:**
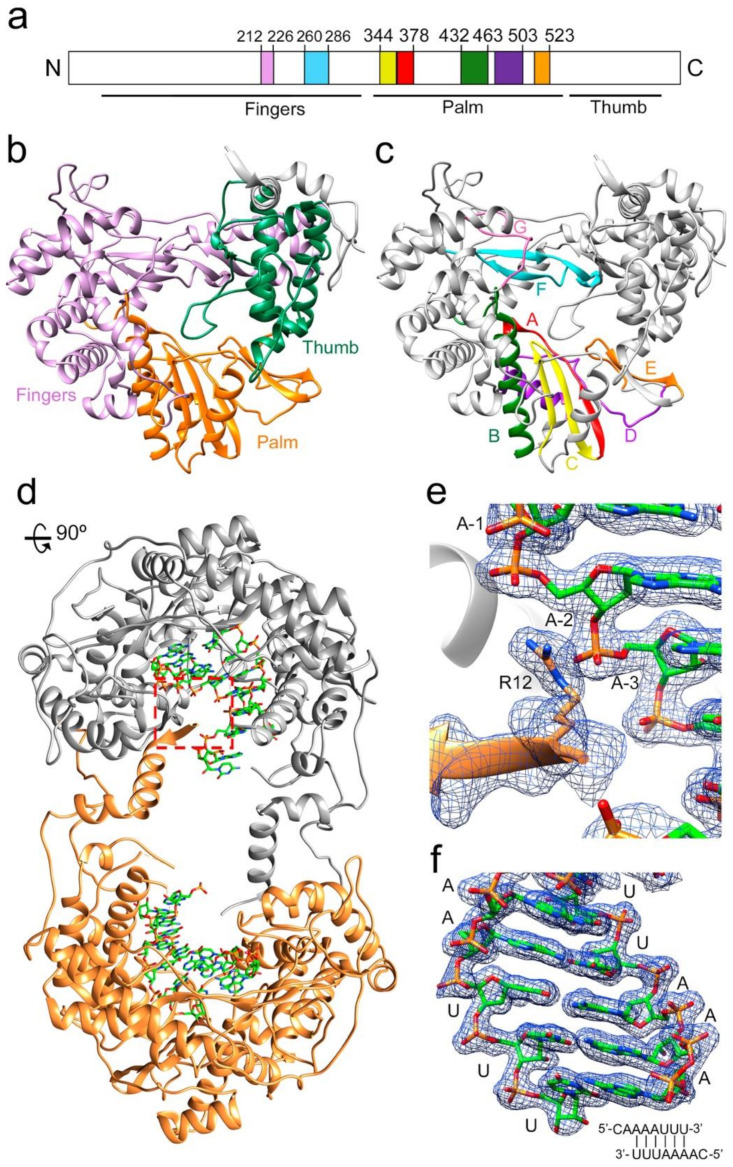
Structure of the TaV RdRP and RdRP-RNA dimers. (**a**) linear representation of TaV_pol_ subdomains (fingers, palm and thumb) and conserved structural motifs. Each motif is highlighted in different colors: G (pink) F (cyan), A(red), B (green), C (yellow), D (purple) and E (orange). (**b**) Ribbon diagram showing the overall architecture of the TaV RdRP with the finger, palm and thumb subdomains shown in different colors: fingers (violet), palm (orange) and thumb (green). (**c**) Ribbon diagram of the TaV enzyme showing the seven conserved motifs colored as in a. (**d**) The TaV RdRP-RNA dimers. The two associated molecules are shown as ribbons in grey and orange, respectively. Amino acids at the N- and C-terminal ends are responsible for stabilizing the dimeric structure of the enzyme that is maintained in all Δ10-TaV_pol_ Δ607-624-RNA complexes analyzed. The RNA molecules bound to each subunit are shown as sticks in the atom type color: C, green; P, orange; O, red; N, blue. (**e**) Close-up of the squared region highlighting the interactions established between the N-terminal residue R12 of one molecule (orange) with the phosphates at positions -2 and -3 of the RNA template bound to the second RdRP molecule. The 2Fo-Fc electron density map around the region is displayed at a contour of 1.5σ (blue mesh). (**f**) The 2Fo-Fc electron density map shown around the dsRNA molecule. The sequence of the template-primer RNA is schematically represented at the panel bottom.

Similar to the apo-form of TaV_pol_ [7], the binary Δ10-TaV_pol_Δ607-624-RNA and ternary Δ10-TaV_pol_Δ607-624-RNA/rNTP complexes were organized in dimers, where the N- and C-terminal arms of each monomer extend out of the polymerase core and are involved in a number of intermolecular interactions that stabilize the dimeric structure (Figure 1a).

**Figure 2:**
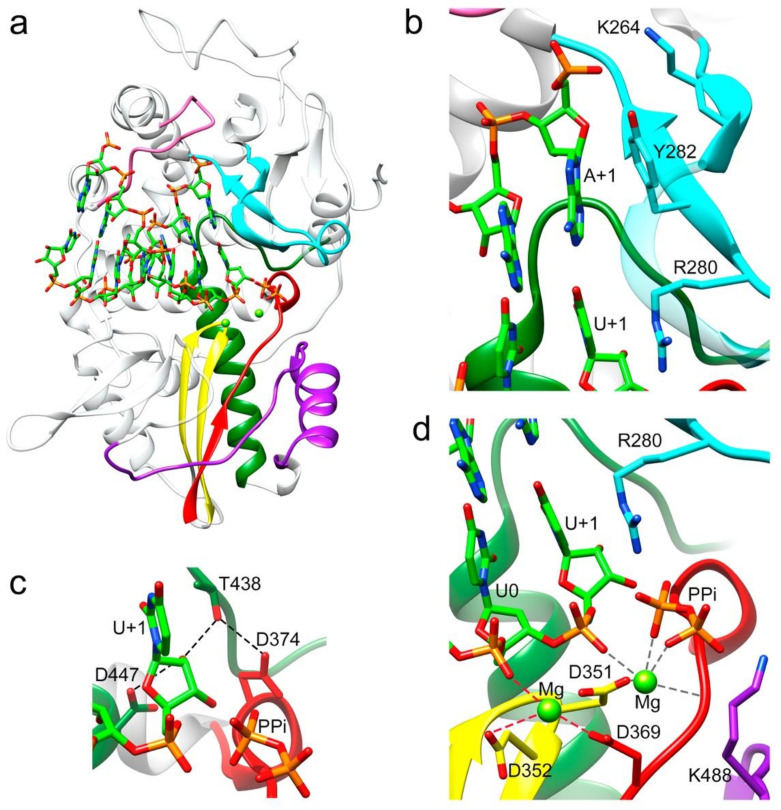
Interactions between the TaV RdRP and the template/primer/product RNA in the RdRP catalytic cavity. (**a**) Side view of the overall structure of the Δ10-TaV_pol_Δ607-624/5′CAAAAUUU3′/UTP complex with the UMP molecule incorporated in a pretranslocation state. The polymerase molecule is shown as grey ribbons, with the conserved structural motifs highlighted in different colors: G (pink) F (cyan), A (red), B (green), C (yellow) and D (purple). The dsRNA molecule bound to the central cavity is represented as sticks in the atom type color: C green, P orange, O red and N blue. Panels b to d show different close up views of the RNA–protein interactions. Molecules are colored as in A and selected protein side chains are represented as sticks and explicitly labeled. (**b**) The template acceptor base A+1 appears to always be stacked to the Y282 side chain, whereas its triphosphate moiety appears salt bridged to K264 in motif F. A second stacking interaction was established between the guanidinium group of R280 (motif F) and the newly incorporated U in position +1. (**c**) The hydrogen bonding network involved in the 2′-hydroxyl recognition of the recently incorporated U+1 nucleotide. Hydrogen bonds are shown as black dashed lines. (**d**) Organization of the metal cluster after nucleotide incorporation into RNA, the product before translocation. Coordination distances < 2.5 Å for Mg^2+^ (B) are shown as dark grey dashed lines. In this structure, Mg^2+^ (A) is loosely coordinated as would be expected of a complex trapped in post-catalysis (distances < 3 Å are shown as red dashed lines). The liberated pyrophosphate (PPi) is present in the catalytic site, coordinated with Mg^2+^ (B) and salt bridged with residue R280.

**Figure 3:**
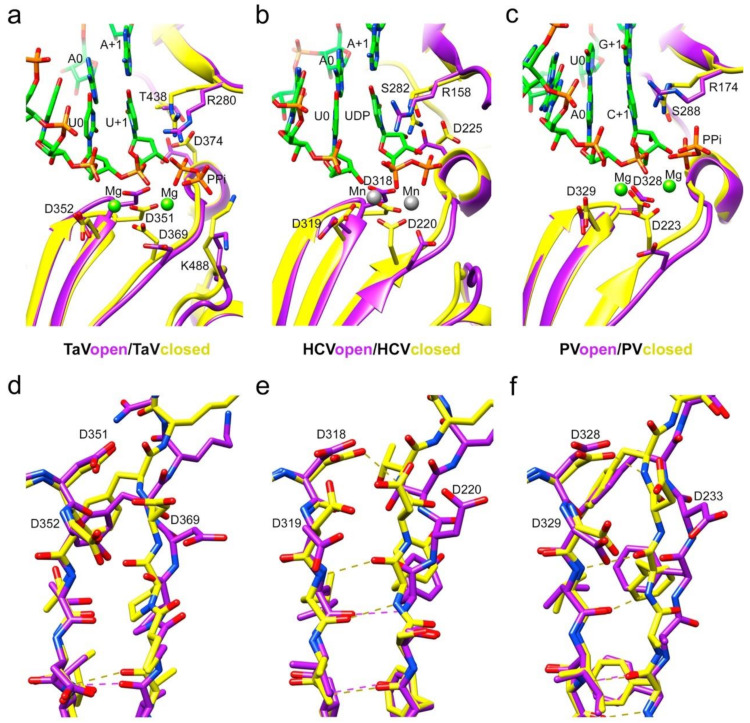
Local rearrangements of the TaV_pol_ active site on substrate binding. Comparison with the open and closed active site conformations in Flavivirus and Picornavirus RdRPs. (**a**) Ribbon representation of the TaV_pol_, apo form, showing an open conformation of the active site (magenta) compared with the TaV_pol_Δ607-624/5′CAAAAUUU3′ complex after UMP incorporation (closed conformation; yellow). Only the base pair A+1:U+1, A0:U0 and A-1:U-1 are shown in the stick representation in the atom type color: C green, P orange, O red and N blue. The pyrophosphate byproduct (PPi), present in the catalytic site, is also shown as sticks. The two Mg+2 ions present in the active site of the TaV_pol_Δ607-624/5′CAAAAUUU3′ complex are shown as green spheres. Changes in the main chain conformation and reorientation of side chains of residues: D351 (active site; motif C), D369 (active site; motif A) and D374 (NTP 2′OH recognition; motif A) and K448 (general acid; motif D) are apparent. (**b**) Ribbon representation of the HCV RdRP (closed, PDB id 4WTA, yellow) in complex with a template/primer RNA. Only the base A+1 and A0 in the template and U0 in the primer are shown as sticks as in A. The incoming UDP substrate (atom type sticks), base paired with A+1 and the two Mn2+ ions (grey spheres) are also shown and explicitly labeled. The HCV open conformation (purple) corresponds to the apo enzyme (PDB id: 2XI2). The side chains of the RNA-interacting residues in the catalytic site, motifs A to D, are shown as sticks and explicitly labeled. (**c**) Ribbon representation of PV the RdRP (PV closed, PDB id: 3OPL7, yellow) in complex with a template/primer/product RNA (with the base pairs G+1:C+1 and A0:U0 shown and explicitly labeled), and two Mg^2+^ ions (green spheres). The PPi byproduct is also shown as sticks. PV open (yellow) corresponds to a PV RdRP–RNA complex after reaction and translocation (PDB id: 3OL6). Panels d to f show stick representations of the superimpositions of the β-strands containing motifs C and A in TaV_pol_ (**d**), HCV (**e**) and PV (**f**), with the main chain hydrogen bonds indicated in dashed lines and the side chains of the catalytic aspartic acid residues explicitly labeled.

The RNA used in the cocrystallization experiments was an octanucleotide (sequence 5′CAAAAUUU 3′), able to form a 6-base pair duplex, flanked by two 2-nucleotide 5′ overhangs, acting as both an RNA template and primer. A self-complementary RNA plus two ssRNA nucleotides of equal sequence was previously employed to trap different elongation complexes of the HCV NS5B RdRP [23]. The final refined electron density for both binary (Figure 1b,c) and ternary complexes was well defined for all template-primer residues forming the dsRNA stretch. The density was also clearly observed in the position of the first overhang nucleotide (A+1) in the template binding channel, becoming weaker in the 5′ end (C+2 nucleotide). In the binary complex, the bound RNA mimics a post-translocation state, ready for nucleotide binding. This is in contrast to that found with the hepatitis C virus RdRP binary complexes obtained by soaking, using RNA template-primers designed as chain terminators [24]. Protein–RNA interactions in the polymerase central cavity are conserved in all structures analyzed. The templating nucleotide A+1 is located above the active site with the adenine base fully stacked on the upstream duplex (Figure 2a,b). The position of the A+1 base is stabilized by stacking interactions with the aromatic side chain of Y282 in the polymerase motif F (Figure 2a). This position of the templating base facilitates direct Watson and Crick pairing with the incoming nucleotide (Figure 2a,b and Figure 3a,b,d,e). In addition, the hydroxyl group of Y282 is hydrogen bonded to oxygen O1 of the A+1 phosphate moiety (Figure 2b). The phosphodiester backbone of A+1 and A0 is also recognized by the amide nitrogen atom of residues S215 (A+1) and T210 (A0) within motif G, and the basic side chains K264 (A+1) and K224 (A0) of motifs F and G, respectively, stabilizing the template RNA towards the active site cavity (Figures S1 and S2 and Table S1). Template phosphates at positions A-1 and A-2 appeared salt bridged with the R12 side chain from the N-terminal arm of the neighboring polymerase in the dimer (Figure 1b and Figure S2 and Table S1). In the binary complex, the 2′hydroxyl group of the uridine at the 3′-end of the primer strand (U0) contacts the main chain residues Y349 and D351 of motif C, and the 3′OH was bridged to the D351 side chain by a water mediated interaction (Figure 2c). In addition, the T444 side chain bound the O2 atom of the U0 base (Figure 2c). All these interactions helped to stabilize the position of the primer nucleotide in an optimal orientation for RNA elongation. Phosphates in the primer strand were mainly recognized by residues R269 in motif F (U-3) and R545 (U-2) and R564 (A-4) of the thumb subdomain (Figures S1 and S2 and Table S1). Other protein–RNA interactions mediated by solvent molecules were also present.

Except for the deletion of the λ6 loop, the overall structure of the Δ10-TaV_pol_Δ607-624 protein bound to RNA was very similar to the unbound TaV_pol_ (root mean square deviation (r.m.s.d) values of 0.5 Å for the superposition of 540 residues), showing that no important rearrangements of the TaV_pol_ conformation were induced by the RNA binding. Noticeable conformational changes, consisting of a main-chain movement of motif A towards motif C and the side chain repositioning of the catalytic D369 (motif A) near the active site, have been observed in all RNA complexes (Figure 3a,b). However, in this “closed” palm conformation, preserved in the three complexes, the motif-C–motif-A β-sheet appeared not to be completed (Figure 3a–c,e).

### 3.3. The Structure of the Δ10-TaV_pol_Δ607-624/RNA/rNTP Ternary Complexes

**Figure 4:**
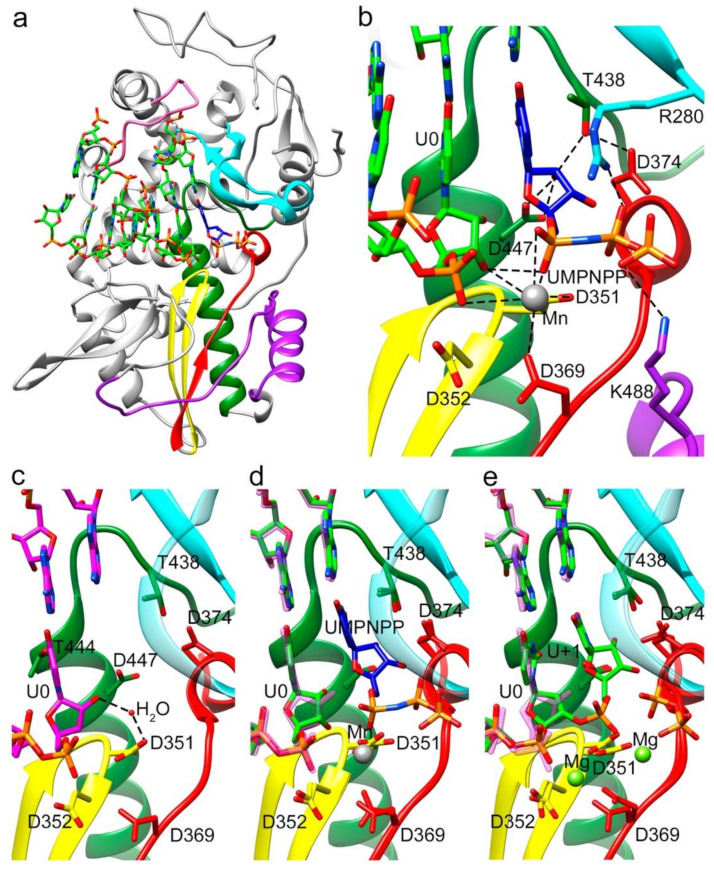
Overall structure of the TaV RdRP in complex with RNA and the incoming nucleoside analogue uridine-5’-[(α,β)-imido]triphosphate (UMPNPP). (**a**) Side view of the overall structure of the Δ10-TaV_pol_Δ607-624/5′CAAAAUUU3′/UMPNPP complex enzyme (grey) with the conserved motifs and the bound RNA template/primer colored as in Figure 1. The substrate analogue is shown as sticks, colored according to atom type (C blue). (**b**) Close up view of the interactions involved in substrate analogue recognition. The side chains of residues D374 (motif A) and T438 and D447 (motif B) that participate in UMPNPP 2′-OH recognition are shown as sticks and explicitly labeled. One Mn^2+^ ion was found in the active site interacting with D351 (motif C), D369 (motif A) and the phosphate 1 moiety of the substrate. (**c**) Close up view of the active site region (same orientation as in B), in the structure of the TaV_pol_Δ607-624/5′CAAAAUUU3′ complex obtained in the absence of incoming nucleotides. The polymerase molecule is shown as green ribbons with the key active site chain depicted as sticks. (**d**) Superimpositions of the TaV_pol_Δ607-624/5′CAAAAUUU3′ complex structures in the absence (RNA carbon atoms in magenta) and in the presence of the UMPNPP analogue (RNA carbon atoms in green; UMPNPP in blue). The Mn^2+^ ion is shown as a grey sphere. (**e**) Superimposition of the TaV_pol_Δ607-624/5′CAAAAUUU3′ complex structures in the absence of the incoming substrate (RNA carbon atoms in magenta) and after UMP incorporation, trapped before translocation (RNA carbon atoms in green). The two bound Mg^2+^ ions are shown as green spheres.

Two different ternary complex cocrystals: Δ10-TaV_pol_Δ607-624-RNA/UTP and Δ10-TaV_pol_Δ607-624-RNA/uridine-5’-[(α,β)-imido]triphosphate were obtained to further characterize different steps in the process of RNA synthesis (Figure 2, Figure 3 and Figure S2). The addition of a non-hydrolysable UTP was used to trap the incoming nucleotide in the active site, before its incorporation (Figure 4a,b and Figure S2 and Table S2). The UTP analog was placed in the active site in an equivalent position to that observed in previous structures of RdRP catalytic complexes [1,23]. In this position, the pyrimidine ring formed a Watson–Crick base pairing with the templating nucleotide A+1. An extensive network of interactions involving the ribose hydroxyl groups 2′ and 3′ were observed, stabilizing the position of the UTP analog in the active site. The side chain of D374 (motif A) was repositioned to contact both the 3′OH group of the ribose moiety and the T438 side chain (motif B). The ribose 2′OH was also recognized by T438 and D447 of motif B (Figure 3b and Figure S2 and Table S2). Despite the addition of the Mg^2+^/Mn^2+^ mixture to the crystals by soaking, only one peak of extra density was identified in the structure to position one ion. This metal ion was located close to the side chain of D351 (motif C) but far away of the triphosphate moiety of the rNTP analog that was salt bridged with the basic side chains K488 (motif D) and R280 of motif F in the rNTP tunnel (Figure 4b).

The incubation of Δ10-TaV_pol_Δ607-624/5′CAAAAUUU3′/UTP complex cocrystals with Mg^2+^ resulted in the hydrolysis of the triphosphate molecule, with the incorporation of UMP into the primer molecule in a pretranslocation state (Figure 2). In this state, the 2′-hydroxyl group appeared hydrogen bonded to side chains D447 (motif B) and T438 (motif B), which in turn was bonded to D374 (motif A) (Figure 2c and Figure S2 and Table S3). Two Mg^2+^ ions occupied the active site, coordinated by residues Asp369 (motif A), Asp351 (Motif C) and the pyrophosphate (PPi) byproduct. The PPi molecule was also salt bridged by K488 (motif D) and R280 (motif F) in the rNTP tunnel (Figure 2d and Figure S2 and Table S4). The newly incorporated uracil (U+1) in this pretranslocation state established a Watson and Crick pair with the A+1 base of the template strand and provided the new 3′ end of the primer terminus.

### 3.4. Non Catalytic Mg^2+^ in the Central Cavity of TaV RdRP

The X-ray analysis of the Δ10-TaV_pol_ construct, crystallized in the presence of large amounts of Mg^2+^ (19% PEG 3350, 0.18 M MgSO_4_), revealed the existence of a second metal ion-binding site located nearby the catalytic cavity, in a non-catalytic position. The resulting electron density maps at 2.07 Å resolution showed strong peaks of extra density in the two molecules of the asymmetric unit. The Mg^2+^ ions were located at 7 Å from the catalytic aspartate D351 (motif C), coordinated by the side chain carboxyl oxygen of D352 (motif C) and five water molecules, in a perfect octahedral geometry (Figure S2). The solvent molecules participating in Mg^2+^ coordination appear also to interact with the polymerase side chains of residues of D369: w1, w2 and w3; T353: w4, with the main chain N and carboxyl oxygens of residues T353 (N), P368 (O)M w4, with the main chain oxygen of K51: w3 and with the main chain oxygen of C3676: w5 (Figure S3). The presence of non-catalytic Mg^2+^ ions at the polymerases central cavity were previously described in other RdRPs [25] and recently, the role of these ions in the nucleotide addition cycle has been reviewed by Gong and collaborators [26]. The position of the noncatalytic Mg^2+^ in Δ10-TaV_pol_ perfectly match that identified in other RdRP structures prior to active site closure [26].

### 3.5. Large Movements in the F Motif

**Figure 5:**
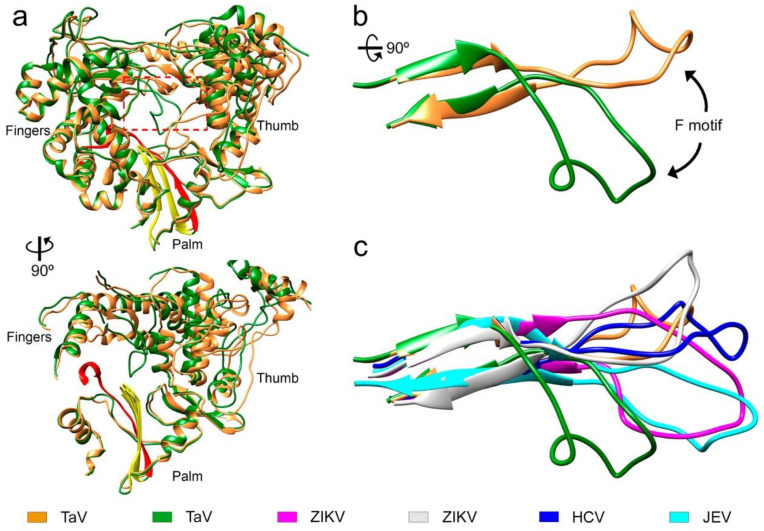
Conformational changes in the motif F of TaV_pol._ (**a**) Front and side views of the overall structure of the TaV RdRP, found both in the unbound-form and in the RNA catalytic complexes (orange ribbons). The structure of a new conformation of the unbound enzyme, exhibiting a large rearrangement of motif F is superimposed (green ribbons) The large movement of motif F is also accompanied by a significant rotation of the thumb subdomain. Structural motif A and C in the palm subdomain are highlighted in red and yellow respectively. (**b**) Close up views of the TaV_pol_ motif F superimposition. (**c**) Structural variability of motif F in TaV_pol_ compared to different members of the Flaviviridae family: HCV (dark blue; PDB id: 4WTA), ZIKV position 1 (grey; PDB id: 6I7P), ZIKV position 2 (magenta; PDB id: 5UOC) and JEV (cyan; PDB id: 4MTV).

In one of the crystals forms from the Δ10-TaV_pol_Δ607-624 construct, obtained in the presence of the RNA oligonucleotide 5′-CAAAAUUU-3′ and soaked with UTP/Mg^2+^, we captured an alternative conformation of motif F in which residues from T267 to T279 move from their expected position in close contact with the thumb domain toward the active site cavity. In this alternative conformation the negatively charged residue D273 that has moved about 20 Å from its original position was located close to the active site, in a position that interferes with the binding of the template-primer RNA (Figure 5, Video S1). This movement of motif F was associated with a significant rearrangement of the orientation of the thumb subdomain relative to the fingers (Figure 5). Structural changes also include a reorientation of the relative disposition of monomers in the dimer, in which one monomer of Δ10-TaV_pol_Δ607-624 (with motif F moved) shows a 25° hinge movement with respect to the same position in the RNA-bound Δ10-TaV_pol_Δ607-624 (Figure S4). Neither the RNA nor the incoming nucleotide were present in the structure despite these substrates being present in the crystallization solution. Similar motif F rearrangements have been described in the structures of the RdRPs of some flaviviruses [13,27].

## 4. Discussion

The TaV RdRP domain was previously structurally and biochemically characterized, showing two essential elements controlling polymerization activity: the N-terminal arm that invades the template binding channel of the neighboring RdRP molecule and the λ6 loop that protrudes towards the catalytic cavity, providing a platform for initial rNTP binding in the de novo replication initiation [7].

The first 10 N-terminal residues were not visible in any of the structures determined and we suspected that they could probably disturb important interactions with the RNA template. As our main aim was to obtain crystallizable TaV_pol_-RNA catalytic complexes we decided to eliminate the first 10 residues of the protein to facilitate the positioning of the RNA template-primer in the central cavity. TaV_pol_ mutant with deletion of the 27 N-terminal residues showed higher polymerase activity than the equivalent construct of wild type TaV_pol_, demonstrating that the catalytic active site was not damaged by deletions in this region [7]. However, we were unable to obtain catalytic TaV_pol_–RNA complex crystals from Δ27-TaV_pol_ or Δ10-TaV_pol_ constructs.

The TaV_pol_ λ6 loop (residues 591–625) is structurally equivalent to the priming loops of flaviviruses and bacteriophage ϕ6 [12,23,24,28]. Indeed, the structure of the TaV_pol_-ssRNA-ATP complex, determined previously, showed the nucleotide substrate resting on loop λ6, with the adenine base stacked to Y611 and F613 side chains [7]. Biochemical data also indicated that TaV_pol_ was able to perform RNA synthesis using a de novo replication initiation mechanism, similar to those found in flaviviruses and in bacteriophage ϕ6 [7,12,29]. However, the initiation platforms block the egress for RNA elongation and important rearrangements would be required in the RdRP structure to move the platform away from the active site, allowing the transition from replication initiation to the elongation states of RNA synthesis [23,24]. In fact, the TaV_pol_ deletion mutant lacking five amino acids from the loop tip, TaV_pol_Δ611–617, showed an increased elongation activity when compared to the wild type enzyme [7]. Comparable increased activities were also observed after similar deletions within the equivalent priming loops of HCV [23,24] and Dengue virus [28] RdRPs. In addition, the high-resolution structures with processive elongation complexes of HCV NS5b have only been obtained from RdRP constructs, containing deletions in the priming loop [23,24]. In this work, we used the Δ10-TaV_pol_Δ607-624 construct to obtain the high resolution structures of a binary Δ10-TaV_pol_Δ607-624-RNA complex (2.18 Å resolution) by cocrystallization and two ternary complexes: Δ10-TaV_pol_Δ607-624-RNA-UTP (3.1 Å resolution) and Δ10-TaV_pol_Δ607-624-RNA-uridine-5’-[(α,β)-imido]triphosphate (2.4 Å resolution) by cocrystallization, followed by soaking with Mg^2+^ (the UTP complex) or Mn^2+^ (the uridine-5’-[(α,β)-imido]triphosphate complex) ions into ternary form cocrystals.

As expected, the shortening of the λ6 loop resulted in an expansion in the polymerase central cavity providing space to accommodate the dsRNA elongation product (Figure 1). Structural comparisons between the apo-form of TaV_pol_ and Δ10TaV_pol_Δ607-624 complexed with the template-primer oligonucleotide showed that no other large rearrangements, additional to λ6 loop elimination, were induced in the polymerase by the RNA binding. A noticeable local change included a main-chain movement of motif A, approaching motif C and the side chain repositioning of the catalytic aspartate of motif A (D369) towards the central cavity, closing the catalytic site (Figure 4a).

The growing biochemical and structural information on replication-elongation complexes, available for different members of (+)ssRNA viruses have provided great insights into the RdRP rearrangements associated to the different steps of the catalytic cycle [1,24,27,30,31,32,33,34,35,36]. Based on the poliovirus PV and Enterovirus 71 EV71 data, Peersen and colleagues proposed that the nucleotide incorporation cycle could be divided into six major states: S1, template-primer binding; S2, NTP binding; S3, active site closure; S4, catalysis; S5 opening of the active site; and S6 translocation and pyrophosphate release [34]. All these steps have been caught in crystal structures, including various translocation intermediates [37,38]. Initially the RdRP binds the RNA template-primer in an open active site conformation, characterized by a partial formation of the central three-stranded β-sheet of the palm subdomain. The initial binding of incoming rNTP occurs also in this polymerase conformation. This rNTP is first probed by base pairing and its final alignment for the catalytic reaction includes a movement of catalytic motif A towards the motif C completing a three-stranded β-sheet [34]. In addition, a side chain movement of the motif A catalytic aspartate allows the positioning of the first metal ion, which together with the second cation, brought in by the rNTP, promotes the nucleophilic attack of the 3′-OH of the primer onto the rNTP α-phosphate. It has been proposed that this motif A movement that also includes a side chain repositioning of a second aspartic acid within the motif A (equivalent to D374 in TaV) (Figure 3a and Figure 4a), leaving space for the 2′OH group of the aligned NTP, represents a fidelity checkpoint in picornavirus RdRPs.

In contrast to that described in enteroviruses, the closure of the TaV_pol_ active site occurred without completing the three-stranded β-sheet formed by motifs A and C (Figure 4). The presence of a proline (P368) in the position immediately before the catalytic D369, exclusive of permutatetraviruses [39] appears to be responsible for the breakage of the hydrogen bond that would complete the closure of the two β-strands (Figure 4a,c).

Although the central β-sheet is not complete, the TaV_pol_ active site appears totally closed, with a D351–D369 distance of 5.5 Å, similar to the closed active site conformations observed in PV (D328–D233 5.6 Å) and HCV (D318–D220 5.7 Å) (Figure 4d–f).

Another difference between the elongation complexes of TaV_pol_ compared to those of enteroviruses is that the active site closure takes place on RNA template-primer binding, before rNTP binding and is maintained during rNTP recognition (Figure 3c,d) but also immediately after nucleotide incorporation (Figure 3d). The incubation of Δ10TaV_pol_Δ607-624-RNA-UTP cocrystals in the presence of Mg^2+^ resulted in the hydrolysis of the triphosphate moiety and incorporation of UMP into the primer molecule, in a pretranslocation conformation. In this state, the conserved catalytic residues D351 (Motif C) and D369 (motif A) still remained coordinated with the two Mg^2+^ ions. The position B Mg^2+^ in turn coordinated the ppi byproduct in the rNTP channel. The conserved residues R280 (motif F) and K488 (motif D) were bound the ppi molecule, opposite to metal ions.

In addition, a stalled Δ10TaV_pol_Δ607-624-RNA-rNTP ternary complex was obtained in the presence of the nucleotide analog uridine-5’-[(α,β)-imido]triphosphate to catch the TaV enzyme in a distinct catalytically relevant conformation (Figure 3). In this complex, the incoming rNTP analogue forms a Watson–Crick interaction with the pairing residue of the template strand and the 2′-OH of its sugar moiety is recognized by an extensive network of hydrogen bonds, including a direct contact with the side chains of the motif B residues T438 and D447 (Figure 2c and Figure 3b). In addition, T438 also interacts with the carboxylic acid of D374 (motif A), which moves away from the nucleotide substrate during binding (Figure 2c and Figure 3b), ensuring selectivity for ribonucleotides, similarly to that previously seen for other RdRPs [1,23,31,34]. Only one metal ion was observed in the catalytic site of this structures (Mn^2+^ A), leaving the metal B position empty (Figure 3b).

Altogether, the structural data presented here define the requirements for processive RNA synthesis by TaV_pol_ during the elongation process. These data also remark the important similarity in mechanistic details between permuted (‘non-canonical’) and canonical RdRPs.

## Figures and Tables

**Table 1:** Summary of the X-ray data collection and model refinement statistics.

	Δ10-TaV_pol_ Mg^2+^	TaV_pol_Δ607-624 RNA	TaV_pol_Δ607-624 UMPNPP Mn^2+^/RNA	TaV_pol_Δ607-624 UTP Mg^2+^/RNA	TaV_pol_Δ607-624 UTP Mg^2+^/RNA
**Data Collection**					
Beamline	XALOC	XALOC	P13	P13	XALOC
Wavelength (Å)	0.9793	0.9789	0.9792	0.9792	0.9793
Resolution range (Å)	75.5-2.07 (2.14-2.07)	48.62-2.18 (2.26-2.18)	56.46-2.4 (2.49-2.40)	59.01-3.1 (3.21-3.1)	47.32-3.0 (3.11-3.00)
Space group	P2_1_2_1_2	P2_1_	P2_1_	P2_1_	C2
Unit Cell *a,b,c* (Å) α, β, γ (◦)					
	134.84, 149.00, 100.37 90.0, 90.0, 90.0	65.64, 204.03, 70.08 90.0, 97.2, 90.0	67.48, 206.27, 115.59 90.0, 90.7, 90.0	67.79, 207.30, 115.98 90.0, 91.1, 90.0	215.15, 74.44, 96.56 90.0, 104.65, 90.0
Total reflections	648698 (68419)	332157 (33960)	1469455 (147126)	398644 (40225)	142101 (14520)
Unique Reflections	116569 (12183)	94404 (9474)	122324 (12216)	57906 (5770)	29693 (2956)
Multiplicity	5.6 (5.6)	3.5 (3.6)	12.0 (12.0)	6.9 (7.0)	4.8 (4.9)
Completeness (%)	94.4 (99.9)	99.03 (98.53)	99.44 (99.58)	99.89 (99.84)	98.2 (99.29)
<I/ σ(I)>	9.00 (1.60)	14.15 (2.53)	17.25 (1.52)	6.79 (1.22)	10.5 (1.5)
R_merge_^§^	0.1474 (1.342)	0.071 (0.682)	0.1305 (1.717)	0.3841 (2.066)	0.143 (0.917)
R_p.i.m._^◊^	0.0669 (0.6082)	0.045 (0.446)	0.0389 (0.5098)	0.1578 (0.8418)	0.109 (0.704)
CC_1/2_	0.995 (0.601)	0.997 (0.737)	0.998 (0.725)	0.951 (0.391)	0.941 (0.514)
**Refinement**					
R_work_^†^	0.1729 (0.2481)	0.2199 (0.3402)	0.2145 (0.3443)	0.2253 (0.3361)	0.2498 (0.3912)
R_free_^‡^	0.2043 (0.2970)	0.2346 (0.3305)	0.2156 (0.3880)	0.2513 (0.3394)	0.2663 (0.3841)
Protein Residues	1308	1264	2518	2516	1233
R.m.s. deviations					
Bond length (Å)	0.008	0.012	0.010	0.006	0.004
Bond Angle (◦)	0.900	1.640	1.390	0.860	0.720
Ramachandran Plot					
Favored (%)	98.38	98.40	97.39	96.46	96.77
Allowed (%)	1.62	1.64	2.61	3.54	3.14
Outliers (%)	0	0	0	0	0.08
PDB ID	7OM2	7OM6	7OM7	7OMA	7OM9

Statistics for the highest-resolution shell are shown in parentheses. ^§^ Rmerge = Σ|I j - <I>| / Σ I j where I j is the intensity of an individual reflection and <I> is the average intensity of that reflection. ^†^ Rwork = Σhkl ||Fobs(hkl)|—|Fcalc(hkl)|| / Σhkl |Fobs(hkl)|, where Fobs and Fcalc are the structure factors, deduced from measured intensities and calculated from the model, respectively. ^‡^ Rfree = as for Rwork but for 5% of the total reflections chosen at random and omitted from refinement. ^◊^ Rpim = Σ hkl √1/n−1Σ|Ii (hkl)−I (hkl)∣ / Σ Σ Ii (hkl).

## Data Availability

The atomic coordinates and structure factors for TaV_pol_ complexes have been deposited in the Protein Data Bank, with the accession codes 7OM2, 7OM6, 7OM7, 7OMA and 7OM9.

## Supplementary Materials

The following are available online at https://www.mdpi.com/article/10.3390/v13071260/s1, Figure S1: Interactions involving the phosphodiester backbone of the RNA template/primer, Figure S2: Diagram of interactions between TaVpol and different ligands, Figure S3: Interactions involving the non-catalytic Mg^2+^, Figure S4: Superimposition of dimeric forms of RNA-bound and motif F moved forms of TaVpol, Supplementary Table S1: contact distances in PDB 7OM6, Supplementary Table S2: contact distances in PDB 7OM7, Supplementary Tables S3 and S4: contact distances in PDB 7OMA. Video S1: Conformational changes in TaV_pol_ induced during movement of motif F.
