# Supplementary material for: Snapshots of a Non-Canonical RdRP in Action
Source: Viruses. 2021 Jun 28;13(7):1260. doi: 10.3390/v13071260 (PMC8310298; doi:10.3390/v13071260)
Supplement: Supplementary file 1 [file viruses-13-01260-s001.zip › viruses-1249960tabele.pdf]

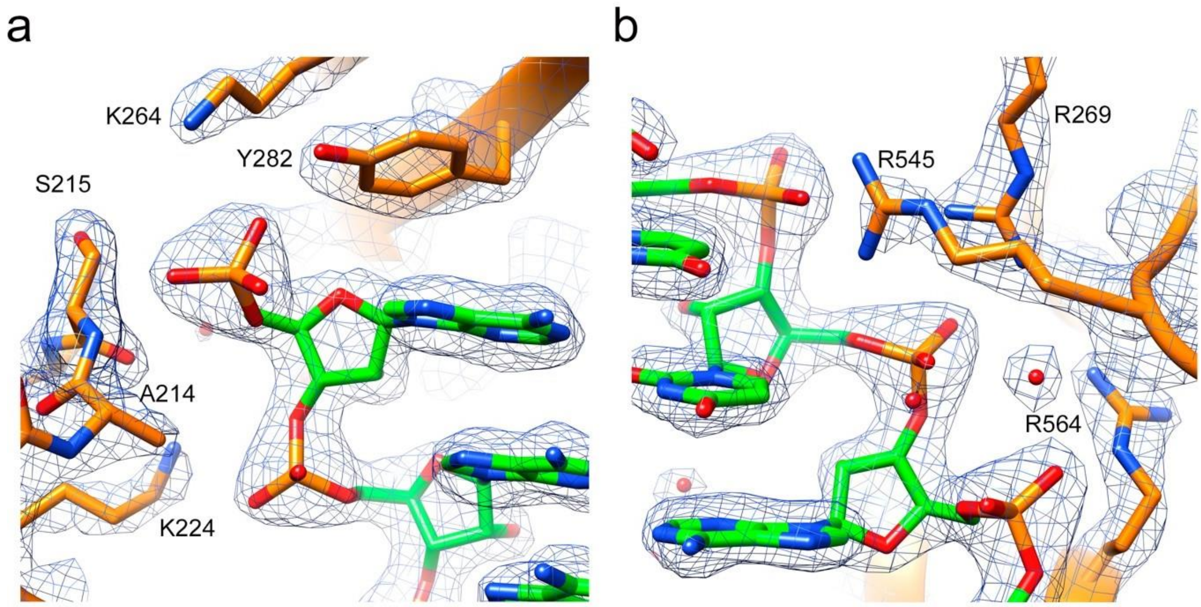

Figure S1. Interactions involving the phosphodiester backbone of the RNA template/primer. (a) Interactions in the template entry channel of TaV_{pol}. Only the nucleotides A+1 and A0 have been represented in contact with the main chain residue S215 of motif G (A+1) and with the basic side chains K264 (A+1) and K224 (A0) of motifs F and G, respectively. (b) The phosphodiester backbone of the primer strand is mainly recognized by residues R269 in motif F(U-3) and R545 (U-2) and R564 (A-4) of the thumb subdomain. The 2Fo-Fc electron density map around the region is displayed at a contour of 1.5σ (cyan mesh).

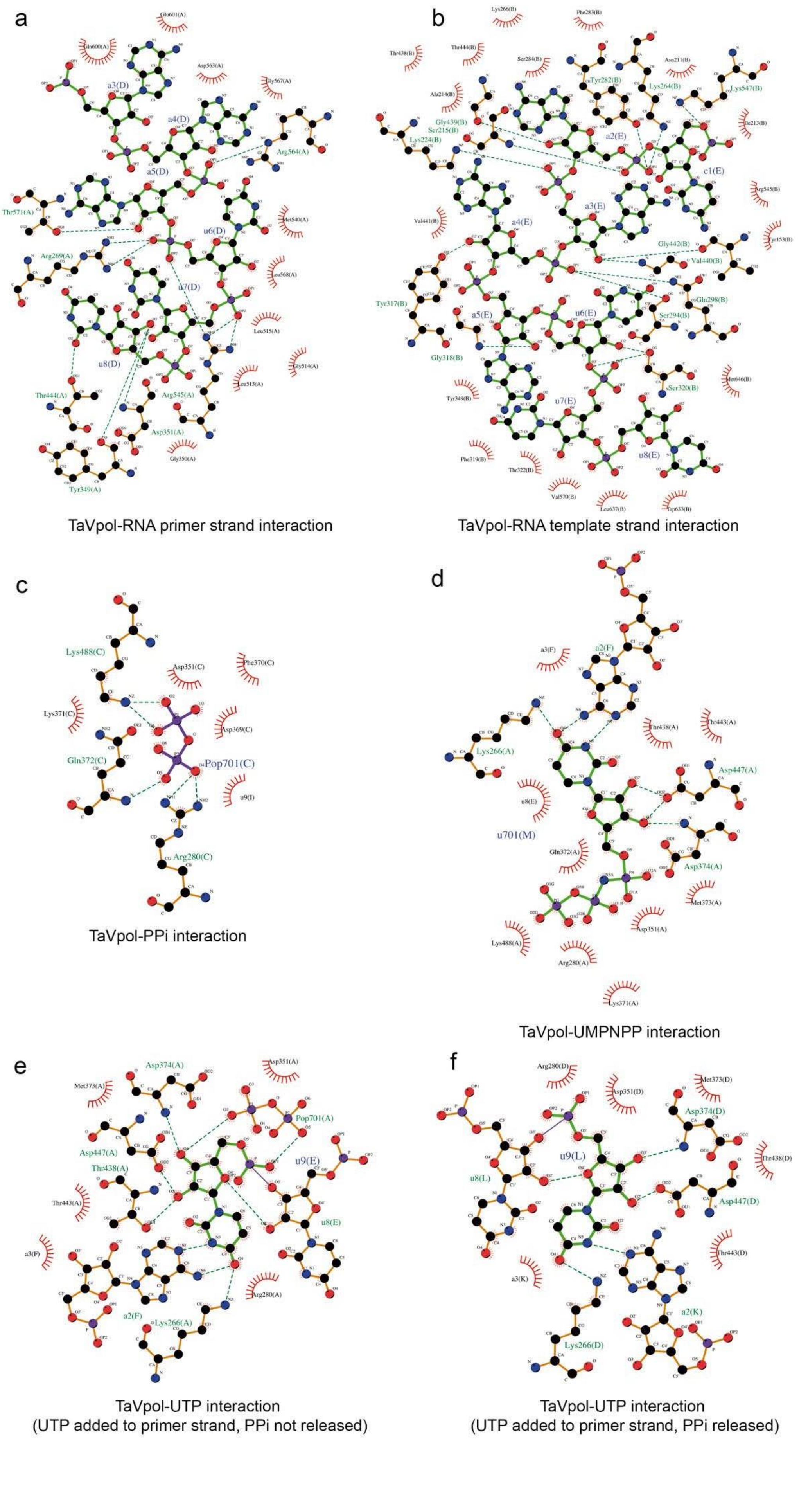

TaVpol-RNA primer strand interaction

TaVpol-RNA template strand interaction

TaVpol-PPi interaction

TaVpol-UMPNPP interaction

TaVpol-UTP interaction
(UTP added to primer strand, PPi not released)

TaVpol-UTP interaction
(UTP added to primer strand, PPi released)

Figure S2. Interaction between TaV_{pol} and the different ligands (a) Contacts between TaV_{pol} and the RNA primer in the binary complex.(b) Contacts between TaV_{pol} and the RNA primer in the binary complex. (c) Interaction involving the pyrophosphate molecule (PPi) in a ternary complex. (d) Interaction with nucleotide analogue UMPNPP. (e) Contacts involving the UMP molecule newly incorporated to the RNA product before ppi release. (f) Contacts involving the UMP molecule newly incorporated to the RNA product before ppi release after PPi. Hydrogen bonds are indicated as dashed lines, hydrophobic contacts are represented by an arc with spokes radiating towards the ligand atoms they contact. The contacted atoms are shown with spokes radiating back. Chain name is indicated in parenthesis and nucleotide residue with lower case letter followed by its position number. Bonds in the RNA chains are depicted in green, phosphate atoms in purple, carbon atoms in black , oxygen atoms in red an nitrogen atoms in blue.

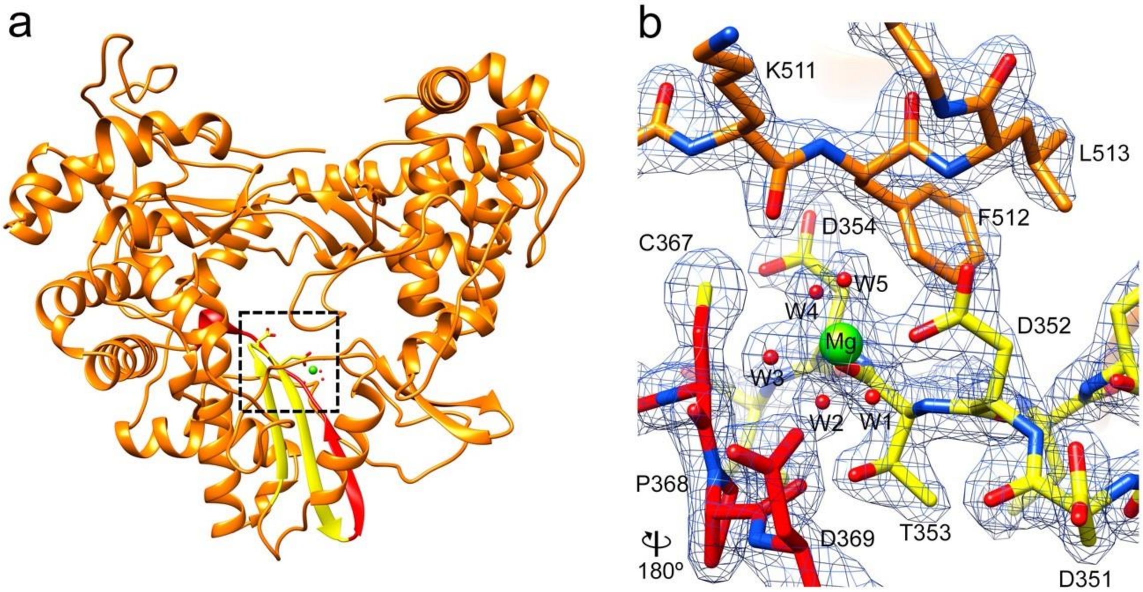

Figure S3. Interactions involving the non-catalytic Mg^{2+}. (A) Front view of the TaV_{pol} structure (orange ribbons), highlighting the central β-sheet of the palm subdomain (yellow and red strands), the motif C aspartates (atom type sticks) and the non-catalytic Mg^{2+} (green sphere). (B) Close up of residues in close contact to the non-catalytic Mg^{2+}.

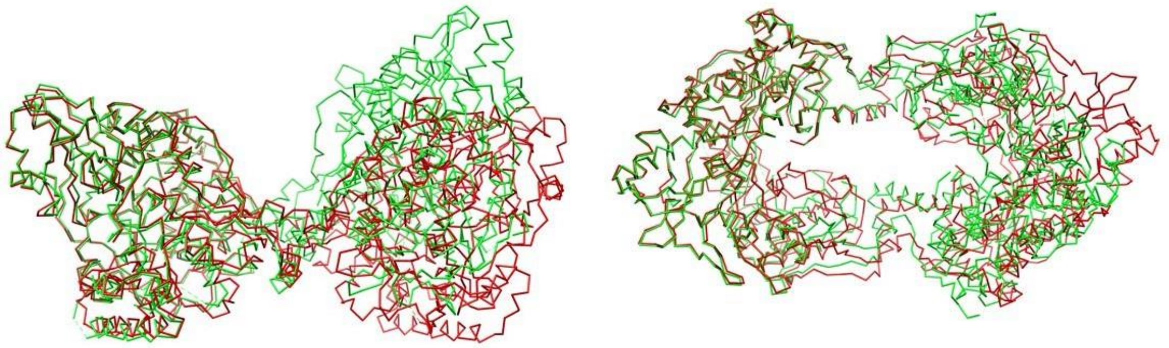

Figure S4. Superimpositions of RNA-bound and motif F-moved forms of Δ10-TaV_{pol}Δ607-624. The RNA-bound form is shown in red and the motif F-moved form of TaV_{pol} in green. One monomer of the motif F-moved form shows a ~25° hinge rotation with respect to the same position in the RNA-bound form.

Table S1. Contact distances between the TaV_{pol} residues and the template-primer RNA in the structure of the binary complex (PDB: 7OM6). The specific atoms involved in contacts are shown in brackets. Only H-bonds and salt bridges are considered.

PDB code		RNA nucleotide [Atom]	Distance (Å)	TaVpol residue [Atom]
7OM6	Primer RNA strand	U7[OP2]	3.87	ARG269[NH1]
		U6[OP1]	3.03	ARG269[NH1]
		U6[OP1]	2.80	ARG269[NH2]
		U8[O3']	3.01	ASP351[N]
		U8[O2]	3.28	THR444[OG1]
		U7[O3′]	3.84	GLY514[N]
		U7[OP2]	3.21	ARG545[NH1]
		U6[O5']	3.19	ARG545[NH2]
		U7[OP2]	3.00	ARG545[NH2]
		U6[OP2]	3.17	ARG545[NH2]
		A5[OP1]	2.94	ARG564[NE]
		U6[O4']	3.64	THR571[OG1]
		A5[O2']	2.74	THR571[OG1]
	Template RNA strand	C1[N4]	3.32	ARG545[O]
		C1[O5']	3.00	LYS547[NZ]
		C1[O4']	3.73	LYS547[NZ]
		C1[O3']	3.68	TYR282[OH]
		A2[OP1]	2.67	TYR282[OH]
		A2[OP1]	3.04	LYS264[NZ]
		A2[OP2]	3.01	SER215[N]
		A2[O3']	3.66	LYS224[NZ]
		A3[OP1]	3.77	THR210[OG1]
		A3[OP1]	2.89	LYS224[NZ]
		A3[O2']	3.19	GLY442[N]
		A3[O2']	3.89	THR443[N]
		A4[OP1]	2.88	GLN298[NE2]
		A4[OP1]	2.77	SER294[OG]
		A4[O3']	3.53	TYR317[OH]
		A4[O2']	2.70	TYR317[OH]
		U6[O2']	2.82	SER320[OG]

Table S2. Description of TaV_{pol} and Uridine-5'-[(α,β)-imido]triphosphate (UMPNPP) contacts mediated by hydrogen bonds.

PDB code	UMPNPP [Atom]	Distance (Å)	TaVpol residue [Atom]
7OM7	UMPNPP 701[O2']	3.45	THR438[OG1]
	UMPNPP 701[O3']	2.99	ASP374[N]
	UMPNPP 701[O4]	2.79	LYS266[NZ]
	UMPNPP 701[O2']	2.96	ASP447[OD2]
	UMPNPP 701[O3']	2.65	ASP447[OD2]

Table S3. Hydrogen bonding contacts between TaV_{pol} and the elongated RNA strand found in the ternary complex, in the post catalytic state. Contacts involving the incorporated UTP(9) are highlighted in blue.

PDB code		RNA nucleotide [Atom]	Distance (Å)	TaVpol residue [Atom]
7OMA	Primer RNA strand	A5[O2']	2.66	THR571[OG1]
		A5[O2']	3.41	LEU568[N]
		U6[OP1]	2.61	ARG269[NH2]
		U6[O4']	3.88	THR571[OG1]
		U7[OP2]	3.54	ARG545[NH1]
		U7[OP2]	3.70	ARG545[NH2]
		U7[O2]	3.14	TYR349[OH]
		U8[O2]	3.52	THR444[OG1]
		U9[OP2]	3.66	ARG280[NH2]
		U9[O3']	3.66	ASP374[N]
		U9[O2']	2.76	THR438[OG1]
	Template RNA strand	C1[N4]	3.74	TYR153[OH]
		C1[OP1]	3.87	LYS264[NZ]
		C1[OP2]	3.58	SER215[N]
		C1[O3']	3.67	TYR282[OH]
		A2[OP1]	2.97	TYR282[OH]
		A2[OP1]	2.78	LYS264[NZ]
		A2[OP2]	2.66	SER215[N]
		A2[O3']	3.76	LYS224[NZ]
		A3[OP1]	2.44	LYS224[NZ]
		A3[OP1]	3.34	THR210[OG1]
		A3[O2']	2.95	GLY442[N]
		A4[OP1]	2.94	SER294[OG]
		A4[OP1]	2.97	GLN298[NE2]
		A4[O3']	3.38	TYR317[OH]
		A4[O2']	2.42	TYR317[OH]
		U6[O2']	2.76	SER320[OG]

Table S4. Polar interactions stablished between the TaV$_{pol}$ residues and the PPi by-product foundin the ternary complex, in the in post catalytic state.

PDB code	Molecule PPi [Atom]	Distance (Å)	TaVpol residue [Atom]
7OMA	POP701[O1]	3.88	GLN372[N]
	POP701[O1]	3.20	LYS488[NZ]
	POP701[O2]	2.96	LYS488[NZ]
	POP701[O4]	2.45	ARG280[NH2]
	POP701[O5]	3.75	ARG164[NH2]
	POP701[O6]	3.63	MET373[N]
	POP701[O]	3.83	ARG280[NH2]
